# Using PPI network autocorrelation in hierarchical multi-label classification trees for gene function prediction

**DOI:** 10.1186/1471-2105-14-285

**Published:** 2013-09-26

**Authors:** Daniela Stojanova, Michelangelo Ceci, Donato Malerba, Saso Dzeroski

**Affiliations:** 1 Department of Knowledge Technologies, Jožef Stefan Institute, Jamova cesta 39, Ljubljana, Slovenia; 2 Jožef Stefan International Postgraduate School, Jamova 39, 1000 Ljubljana, Slovenia; 3 Dipartimento di Informatica, Università degli Studi di Bari "Aldo Moro", via Orabona 4, Bari, Italy; 4 Centre of Excellence for Integrated Approaches in Chemistry and Biology of Proteins, Jamova 39, 1000 Ljubljana, Slovenia

## Abstract

**Background:**

Ontologies and catalogs of gene functions, such as the Gene Ontology (GO) and MIPS-FUN, assume that functional classes are organized hierarchically, that is, general functions include more specific ones. This has recently motivated the development of several machine learning algorithms for gene function prediction that leverages on this hierarchical organization where instances may belong to multiple classes. In addition, it is possible to exploit relationships among examples, since it is plausible that related genes tend to share functional annotations. Although these relationships have been identified and extensively studied in the area of protein-protein interaction (PPI) networks, they have not received much attention in hierarchical and multi-class gene function prediction. Relations between genes introduce autocorrelation in functional annotations and violate the assumption that instances are independently and identically distributed (i.i.d.), which underlines most machine learning algorithms. Although the explicit consideration of these relations brings additional complexity to the learning process, we expect substantial benefits in predictive accuracy of learned classifiers.

**Results:**

This article demonstrates the benefits (in terms of predictive accuracy) of considering autocorrelation in multi-class gene function prediction. We develop a tree-based algorithm for considering network autocorrelation in the setting of Hierarchical Multi-label Classification (HMC). We empirically evaluate the proposed algorithm, called NHMC (Network Hierarchical Multi-label Classification), on 12 yeast datasets using each of the MIPS-FUN and GO annotation schemes and exploiting 2 different PPI networks. The results clearly show that taking autocorrelation into account improves the predictive performance of the learned models for predicting gene function.

**Conclusions:**

Our newly developed method for HMC takes into account network information in the learning phase: When used for gene function prediction in the context of PPI networks, the explicit consideration of network autocorrelation increases the predictive performance of the learned models. Overall, we found that this holds for different gene features/ descriptions, functional annotation schemes, and PPI networks: Best results are achieved when the PPI network is dense and contains a large proportion of function-relevant interactions.

## Background

### Introduction

In the era of high-throughput computational biology, discovering the biological functions of the genes/proteins within an organism is a central goal. Many studies have applied machine learning to infer functional properties of proteins, or directly predict one or more functions for unknown proteins [1-3]. The prediction of multiple biological functions with a single model, by using learning methods for multi-label prediction, has made considerable progress in recent years [3].

**Figure 1:**
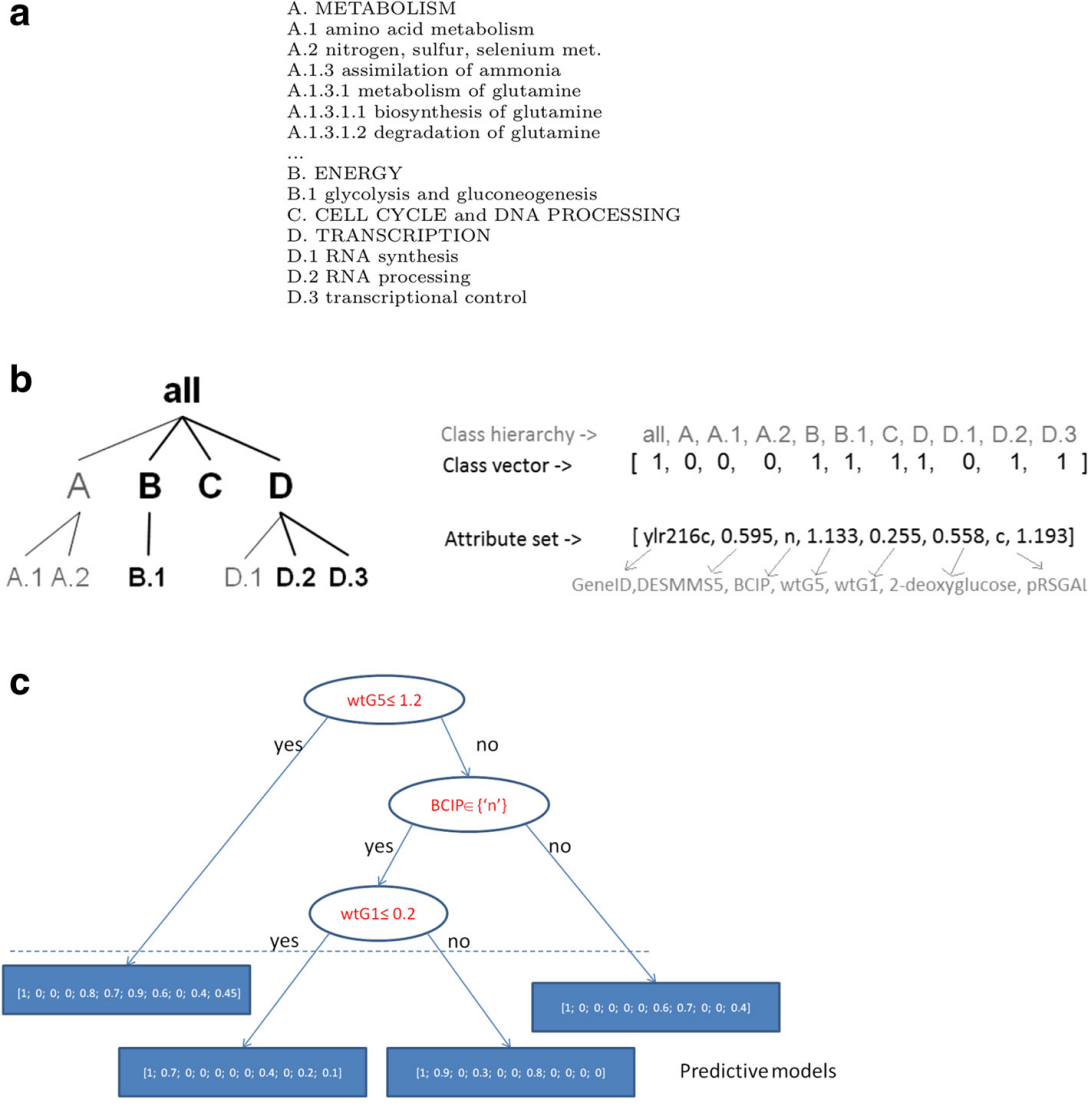
**Example of a hierarchy.** **(a)** A part of the FUN hierarchy [7]. **(b)** An example of input data: The FUN class hierarchy of an example and corresponding class vector and attribute set. **(c)** An example of a predictive clustering tree for HMC. The internal nodes contain tests on attribute values and the leaves vectors of probabilities associated with the class values.

A major step forward is the learning of models which take into account the possible structural relationships among functional classes [4,5]. This is motivated by the presence of ontologies and catalogs such as Gene Ontology (GO) [6] and MIPS-FUN (FUN henceforth) [7], which are organized hierarchically (and, possibly, in the form of Direct Acyclic Graphs (DAGs), where classes may have multiple parents), where general functions include other more specific functions (see Figure 1(a)). In this context, the *hierarchial constraint* must be observed: A gene annotated with a function must be annotated with all the ancestor functions from the hierarchy. In order to tackle this problem, hierarchical multi-label classifiers, that are able to take the hierarchical organization of the classes into account during both the learning and the prediction phase, have been recently used [8].

The topic of using protein-protein interaction (PPI) networks in the identification and prediction of protein functions has attracted increasing attention in recent years. The motivation for this stream of research is best summarized by the statement that "when two proteins are found to interact in a high throughput assay, we also tend to use this as evidence of functional linkage" [5]. As a confirmation, numerous studies have demonstrated the *guilt-by-association* (GBA) principle, which states that proteins sharing similar functional annotations tend to interact more frequently than proteins which do not share them. Interactions reflect the relation or dependence between proteins. In the context of networks of such interactions, gene functions show some form of *autocorrelation*[9].

While *correlation* denotes any statistical relationship between two different variables (properties) of the same objects (in a collection of independently selected objects), *autocorrelation* denotes the statistical relationships between the same variable (e.g., protein function) on different but related (dependent) objects (e.g., interacting proteins). Although autocorrelation has never been investigated in the context of Hierarchical Multi-label Classification (HMC), it is not a new phenomenon in protein studies. For example, it has been used for predicting protein properties using sequence-derived structural and physicochemical features of protein sequences [10]. In this work, we introduce a definition of autocorrelation for the case of HMC and propose a method that leverages on it for improving the accuracy of gene function prediction.

### Motivation and contributions

The method developed in this work, named NHMC, addresses the task of hierarchical multi-label classification where, in addition to attributes describing the genes, such as microarray-derived expression values, phenotype and sequence data, the network autocorrelation of the class values (gene functions) is also considered. The main goal is gene function prediction in the context of gene interaction networks, where network autocorrelation exists among the functional annotations of genes. Each of the aspects of NHMC and network autocorrelation have been addressed individually in the framework of predictive clustering and in particular within the task of learning predictive clustering trees (PCT) [11]. Vens et al. [4] proposed CLUS-HMC, an approach for building PCT for HMC. Stojanova et al. [12] proposed NCLUS, an approach for building PCT to perform regression on network data, taking into account the network autocorrelation of the real-valued (dependent) response variable. We bring both of these recent developments under the same roof and propose NHMC, an approach for building PCT to perform HMC on network data, taking into account the (PPI) network autocorrelation of the hierarchical annotations (of gene functions).

The consideration of network autocorrelation itself raises several challenges. The existence of autocorrelation violates the assumption that instances (in our case genes) are independently and identically distributed (i.i.d.), which underlines most machine learning algorithms. The violation of the i.i.d. assumption has been identified as one of the main reasons responsible for the poor performance of traditional methods in machine learning [13]. Moreover, most of the learning methods which model autocorrelation in networked data assume its stationarity [14]. This means that possible significant variations of autocorrelation throughout the network due to a different underlying latent structure cannot be properly represented.

The consideration of hierarchical multi-label classification introduces additional complications. Network autocorrelation in the context of different effects of autocorrelation can be expected for different class labels. Furthermore, the classes at the lower levels of the hierarchy will have a higher fragmentation: For those classes, the autocorrelation phenomenon will likely be local (or more local than for their ancestor classes). Thus, in HMC tasks, we will need to consider autocorrelation by modeling (and exploiting) its non-stationarity.

While the simultaneous consideration of the relationships among class labels (gene functions) and instances (genes) introduces additional complexity to the learning process, it also has the potential to bring substantial benefits. The method NHMC that we propose will be able to consider gene function hierarchies in the form of DAG structures, where a class may have multiple parents, and to consistently combine two sources of information (hierarchical collections of functional class definitions and PPI networks). In this way, we will be able to obtain gene function predictions consistent with the network structure and improve the predictive capability of the learned models. We will also be able to capture the non-stationary effect of autocorrelation at different levels of the hierarchy and in different parts of the networks.

In this article, we first define the concept of autocorrelation in the HMC setting and introduce an appropriate autocorrelation measure. We then introduce the NHMC algorithm for HMC, which takes this kind of autocorrelation into account. Like CLUS-HMC, NHMC exploits the hierarchical organization of class labels (gene functions), which can have the form of a tree or a direct acyclic graph (DAG). Like NCLUS, NHMC explicitly considers non-stationary autocorrelation when building PCT for HMC from real world (PPI) network data: The models it builds adapt to local properties of the data, providing, at the same time, predictions that are smoothed to capture local network regularities. Finally, we evaluate the performance of NHMC on many datasets along a number of dimensions: These include the gene descriptions, the functional annotation hierarchies and the PPI networks considered.

## Methods

In this section, we introduce the method NHMC (Network CLUS-HMC; a preliminary version of NHMC has been presented in [15]), the major contribution of the paper. NHMC builds autocorrelation-aware models (trees) for HMC. We shall start with a brief description of the algorithm CLUS-HMC, which builds trees for HMC and is the starting point for developing NHMC.

For the HMC task, the input is a dataset *U* consisting of instances (examples) that have the form *u*_*i*_ = (*x*_*i*_,*y*_*i*_) ∈ **X** × 2^*C*^, where **X** = *X*_1_ × *X*_2_…× *X*_*m*_ is the space spanned by *m* attributes or features (either continuous or categorical), while 2^*C*^ is the power set of *C* = {*c*_1_,…,*c*_*K*_}, the set of all possible class labels. *C* is hierarchically organized with respect to a partial order ≼ which represents the superclass relationship. Note that each *y*_*i*_ satisfies the *hierarchical constraint*:

(1) $$c\in y_{i}\Rightarrow \forall c^{\prime }≼c:c^{\prime }\in y_{i}.$$

The method we propose (NHMC) builds a generalized form of decision trees and is set in the Predictive Clustering (PC) framework [11]. The PC framework views a decision tree as a hierarchy of clusters: the top-node corresponds to one cluster containing all the data, that is recursively partitioned into smaller clusters when moving down the tree. Such a tree is called a predictive clustering tree (PCT). PCT combines elements from both prediction and clustering. As in clustering, clusters of data points that are similar to each other are identified, but, in addition, a predictive model is also associated to each cluster. This predictive model provides a prediction for the target property of new examples that are recognized to belong to the cluster. In addition, besides the clusters themselves, PC approaches also provide symbolic descriptions of the constructed (hierarchically organized) clusters.

The original PC framework is implemented in the CLUS system [11] ( http://sourceforge.net/projects/clus/), which can learn both PCT and predictive clustering rules. The induction of PCT is not very different than the induction of standard decision trees (as performed, e.g., by the C4.5 algorithm [16]). The algorithm takes as input a set of training instances and searches for the best acceptable test to put in a node and split the data. If such a test can be found, then the algorithm creates a new internal node labeled with the test and calls itself recursively to construct a subtree for each subset (cluster) in the partition induced by the test on the training instances.

### CLUS-HMC

The CLUS-HMC [4] algorithm builds HMC trees, PCT for hierarchial multi-label classification (see Figure 1(c) for an example of an HMC tree). These are very similar to classification trees, but each leaf predicts a hierarchy of class labels rather than a single label. CLUS-HMC builds the trees in a top-down fashion and the outline of the algorithm is very similar to that of top-down decision tree induction algorithms (see the CLUS-HMC pseudo-code in Additional file 1). The main differences are in the search heuristics and in the way predictions are made. For the sake of completeness both aspects are reported in the following. Additional details on CLUS-HMC are given by Vens et al. [4].

#### Search heuristics

To select the best test in an internal node of the tree, the algorithm scores the possible tests according to the reduction in variance (defined below) induced on the set *U* of examples associated to the node. In CLUS-HMC, the variance of class labels across a set of examples *U* is defined as follows:

(2) $$\text{Var}(U)=\frac{1}{\left|U\right|}\cdot \underset{u_{i}\in U}{\sum }d{\left(L_{i},\bar{L}\right)}^{2},$$

where *L*_*i*_ is the vector associated to the class labels of example *u*_*i*_ (each element of *L*_*i*_ is binary and represents the presence/absence of a class label for *u*_*i*_), $\bar{L}$ is the average of all *L*_*i*_ vectors corresponding to the class labels of examples in *U* and *d*(·,·) is a distance function on such vectors. The basic idea behind the use of the variance reduction is to minimize intra-cluster variance.

In the HMC context, class labels at higher levels of the annotation hierarchy are more important than class labels at lower levels. This is reflected in the distance measure used in the above formula, which is a weighted Euclidean distance:

(3) $$d(L_{1},L_{2})=\sqrt{\overset{K}{\underset{k=1}{\sum }}\omega (c_{k})\cdot {\left(L_{1,k}-L_{2,k}\right)}^{2}}$$

where *L*_*i*,*k*_ is the *k*-th component of the class vector *L*_*i*_ and the class weights *ω*(*c*_*k*_) associated with the labels decrease with the depth of the class in the hierarchy. More precisely, *ω*(*c*) = *ω*_0_·*avg*_*j*_ {*ω*(*p*_*j*_(*c*))}, where *p*_*j*_(*c*) denotes the *j*-th parent of class *c* and 0 < *ω*_0_ < 1). This definition of the weights allows us to take into account a hierarchy of classes, structured as a tree and DAG (multiple parents of a single label).

For instance, consider the small hierarchy^a^ in Figure 1(b), and two examples (*x*_1_,*y*_1_) and (*x*_2_,*y*_2_), where *y*_1_ = {*all*,*B*,*B*.1,*C*,*D*,*D*.2,*D*.3} and *y*_2_ = {*all*,*A*,*D*,*D*.2,*D*.3}. The class vectors for *y*_1_ and *y*_2_ are: *L*_1_ = [ 1,0,0,0,1,1,1,1,0,1,1] and *L*_2_ = [ 1,1,0,0,0,0,0,1,0,1,1]. The distance between the two class vectors is then:

(4) $$\begin{array}{l} d([1,0,0,0,1,1,1,1,0,1,1],[1,1,0,0,0,0,0,1,0,1,1])\\ \;=\sqrt{3\cdot w_{0}^{2}+4\cdot w_{0}^{3}} \end{array}$$

At each node of the tree, the test that maximizes the variance reduction is selected. This is expected to maximize cluster homogeneity with respect to the target variable and improve the predictive performance of the tree. If no test can be found that significantly reduces variance (as measured by a statistical F-test), then the algorithm creates a leaf and labels it with a prediction, which can consist of multiple hierarchically organized labels.

#### Predictions

A classification tree typically associates a leaf with the "majority class", i.e., the label most appearing in the training examples at the leaf. This label is later used for prediction purposes when a test case reaches that leaf. However, in the case of HMC, where an example may have multiple classes, the notion of "majority class" cannot be straightforwardly applied. In fact, CLUS-HMC associates the leaf with the mean $\bar{L}$ of the class vectors of the examples in the leaf. The value at the *k*-th component of $\bar{L}$ is interpreted as the membership score of class *c*_*k*_, i.e., the probability that an example arriving at the leaf will be labeled with a class *c*_*k*_.

For an example arriving at a leaf, binary predictions for each class label can be obtained by applying a user defined threshold *τ* on this probability: If the *i*-th component of $\bar{L}$ is above *τ* (> *τ*), then the leaf predicts the class *c*_*i*_. To ensure that the predictions satisfy the hierarchical constraint, i.e., whenever a class is predicted, its super-classes are also predicted, it suffices to choose *τ*_*i*_ ≤ *τ*_*j*_ whenever *c*_*j*_ is ancestor of *c*_*i*_.

### NHMC

We first discuss the network setting that we consider in this paper. We then propose a new network autocorrelation measure for HMC tasks. Subsequently, we describe the CLUS-HMC algorithm for learning HMC trees and introduce its extension NHMC (i.e., Network CLUS-HMC), which takes into account the network autocorrelation (coming from PPI networks) when learning trees for HMC.

#### Network setting for HMC

Some uses of a PPI network in learning gene function prediction models include: treating the interactions between pairs of genes as descriptive attributes (e.g., binary attributes [17]) and generating new features as combinations of PPI data with other descriptive attributes. Both approaches require that data are pre-processed before applying a network oblivious learning method (e.g., CLUS-HMC). However, the applicability of predictive models built in this way is strongly dependent on PPI network information being available for the testing data, i.e., for the proteins whose gene function we want to predict.

In order to learn general models, which can be used to make predictions for any test set, we use protein interactions as a form of background knowledge and exploit them only in the learning phase. More specifically, in the training phase, both gene properties and network structure are considered. In the testing phase, only gene properties are considered and the network structure is disregarded. This key feature of the proposed solution is especially attractive when function prediction concerns new genes, for which interactions with other genes are not known or are still to be confirmed.

Following Steinhaeuser et al. [18], we view a training set as a single network of labeled nodes. Formally, the network is defined as an undirected edge-weighted graph *G* = (*V*,*E*), where *V* is the set of labeled *nodes*, and $E\subseteq \{\langle u,v,w\rangle |u,v\in V,w\in R^{+}\}$ is the set of *edges*. Each edge *u* ↔ *v* is assigned with a non-negative real number *w*, called the *weight* of the edge. It can be represented by a symmetric adjacency matrix **W**, whose entries are positive (*w*_*ij*_ > 0) if there is an edge connecting *i* to *j* in *G*, and zero (*w*_*ij*_ = 0) otherwise. In PPI networks, edge weights can express the strength of the interactions between proteins. Although the proposed method works with any non-negative weight values, in our experiments we mainly focus on binary (0/1) weights.

Each node of the network is associated with an example pair *u*_*i*_ = (*x*_*i*_,*y*_*i*_) ∈ **X** × 2^*C*^, where $y_{i}=(y_{i_{1}},y_{i_{2}},\ldots ,y_{i_{q}}),q\leq K$, is subject to the hierarchical constraint. Given a network *G* = (*V*,*E*) and a function *η* : *V* ↦ (**X** × 2^*C*^) which associates each node with the corresponding example, we interpret the task of hierarchical multi-label classification as building a PCT which represents a multi-dimensional predictive function *f* : **X** ↦ 2^*C*^ that satisfies the hierarchical constraint, maximizes the autocorrelation of the observed classes *y*_*i*_ for the network *G*, and minimizes the prediction error on *y*_*i*_ for the training data *η*(*V*).

#### Network autocorrelation for HMC

An illustration of the concept of network autocorrelation for HMC is a special case of network autocorrelation [19]. It can be defined as the statistical relationship between the observations of a variable (e.g., protein function) on distinct but related (connected) nodes in a network (e.g., interacting proteins). In HMC, domain values of the variable form a hierarchy, such as the GO hierarchy for protein functions. Therefore, it is possible to define network autocorrelation for individual nodes and for various levels of the hierarchy.

In predictive modeling, network autocorrelation can be a problem, since the i.i.d. assumption is violated, but also an opportunity, if it is properly considered in the model. This is particularly true for the task of hierarchical multi-label classification considered in this work. Indeed, due to non-stationary autocorrelation, PPI network data can provide useful (and diverse) information for each single class at each level of the hierarchy. Intuitively, genes belonging to classes at higher levels of the hierarchy tend to participate in very general types of interactions, while genes belonging to classes at lower levels of the hierarchy tend to participate in very specific and localized interactions. In any case, the effect of autocorrelation changes from level to level (this aspect is also mentioned by Gillis and Pavlidis [20]). For this reason, we explicitly measure autocorrelation and we build a model such that its value is maximized.

#### Geary’s *C* for HMC

In order to measure the autocorrelation of the response variable *Y* in the network setting for HMC, we propose a new statistic, named *A*_*Y*_(*U*), whose definition draws inspiration from Global Geary’s *C*[21]. Global Geary’s *C* is a measure of spatial autocorrelation for a continuous variable. Its basic definition (used in spatial data analysis [22]) is given in Additional file 2.

Let *u*_*i*_ = (*x*_*i*_,*y*_*i*_) ∈ *U* ⊆ **X** × 2^*C*^ be an example pair in a training set *U* of *N* examples. Let *K* be the number of classes in *C*, possibly defining a hierarchy. We represent *y*_*i*_ as a binary vector *L*_*i*_ of size *K*, such that *L*_*i*,*k*_ = 1 if *c*_*k*_ ∈ *y*_*i*_ and *L*_*i*,*k*_ = 0 otherwise, and each *L*_*i*_ satisfies the hierarchical constraint. Let *d*(*L*_*i*_,*L*_*j*_) be a distance measure defined for two binary vectors associated to two examples *u*_*i*_ = (*x*_*i*_,*y*_*i*_) and *u*_*j*_ = (*x*_*j*_,*y*_*j*_), which takes the class-label hierarchy into account.

The network autocorrelation measure *A*_*Y*_(*U*), based on Geary’s *C*, is defined as follows:

(5) $$A_{Y}(U)=1-\frac{(N-1)\cdot \sum_{i}\sum_{j}w_{\text{ij}}\cdot d{\left(L_{i},L_{j}\right)}^{2}}{4\cdot \sum_{i}\sum_{j}w_{\text{ij}}\;\cdot \;\sum_{i}d{\left(L_{i},\bar{L}\right)}^{2}}$$

where $\bar{L}$ is the vector representation of the mean vector computed on all binary vectors associated to example pairs in *U*. The constant 4 in the denominator is included for scaling purposes. The new autocorrelation measure *A*_*Y*_(*U*) takes values in the unit interval [0,1], where 1 (0) means strong positive (negative) autocorrelation and 0.5 means no autocorrelation.

#### The Algorithm

We can now proceed to describe the top-down induction algorithm for building Network HMC trees. The main differece with respect to CLUS-HMC is that the heuristic is different. The network is considered as background knowledge and exploited only in the learning phase. Below, we first give an outline of the algorithm, before giving details on the new search heuristics, which takes autocorrelation into account. We discuss how the new search heuristics can be computed efficiently.

##### 

**Outline of the algorithm** The top-down induction algorithm for building PCT for HMC from network data is given below (Algorithm 1). It takes as input the network *G* = (*V*,*E*) and the corresponding HMC dataset *U*, obtained by applying *η* : *V* ↦ **X** × 2^*C*^ to the vertices of the network.

In practice, this means that for each gene *u*_*i*_ (see Figure 1(b)) there is a set of (discrete and continuous) attributes describing different aspects of the genes. For the experiments with the yeast genome, these include sequence statistics, phenotype, secondary structure, homology, and expression data (see next Section) and a class vector, *L*_*i*_ i.e., functional annotations associated to it.

The algorithm recursively partitions *U* until a stopping criterion is satisfied (Algorithm 1 line 2). Since the implementation of this algorithm is based on the implementation of the CLUS-HMC algorithm, we call this algorithm NHMC (Network CLUS-HMC).

##### 

**Search space** As in CLUS-HMC, for each internal node of the tree, the best split is selected by considering all available attributes. Let *X*_*i*_ ∈ {*X*_1_,…,*X*_*m*_} be an attribute and ${\text{Dom}}_{X_{i}}$ its active domain. A split can partition the current sample space *D* according to a test of the form *X*_*i*_ ∈ *B*, where $B\subseteq {\text{Dom}}_{X_{i}}$. This means that *D* is partitioned into two sets, *D*_1_ and *D*_2_, on the basis of the value of *X*_*i*_.

For continuous attributes, possible tests are of the form *X* ≤ *β*. For discrete attributes, they are of the form $X\in \{a_{i_{1}},a_{i_{2}},\ldots ,a_{i_{o}}\}$ (where $\left\{a_{i_{1}},a_{i_{2}},\ldots ,a_{i_{o}}\right\}$ is a non-empty subset of the domain *Dom*_*X*_ of *X*). In the former case, possible values of *β* are determined by sorting the distinct values in *D*, then considering the midpoints between pairs of consecutive values. For *b* distinct values, *b*-1 thresholds are considered. When selecting a subset of values for a discrete attribute, CLUS-HMC relies on the non-optimal greedy strategy proposed by Mehta et al. [23].

##### 

**Heuristics** The major difference between NHMC and CLUS-HMC is in the heuristics they use for the evaluation of each possible split. The variance reduction heuristics employed in CLUS-HMC (Additional file 1) aims at finding accurate models, since it considers the homogeneity in the values of the target variables and reduces the error on the training data. However, it does not consider the dependencies of the target variables values between related examples and therefore neglects the possible presence of autocorrelation in the training data. To address this issue, we introduced network autocorrelation in the search heuristic and combined it with the variance reduction to obtain a new heuristics (Algorithm 1).

More formally, the NHMC heuristics is a linear combination of the average autocorrelation measure *A*_*Y*_(·) (first term) and variance reduction *Var*(·) (second term):

(6) $$\begin{array}{ll} \;h & =\alpha \cdot \left(\frac{\left|U_{1}|\cdot A_{Y}(U_{1})+|U_{2}|\cdot A_{Y}(U_{2}\right)}{\left|U\right|}\right)\\ & \;+(1-\alpha )\cdot \left({\text{Var}}^{\prime }(U)-\frac{\left|U_{1}|\cdot {\text{Var}}^{\prime }(U_{1})+|U_{2}|\cdot {\text{Var}}^{\prime }(U_{2}\right)}{\left|U\right|}\right) \end{array}$$

where *Var*^′^(*U*) is the min-max normalization of *Var*(*U*), required to keep the values of the linear combination in the unit interval [0,1], that is:

(7) $${\text{Var}}^{\prime }(U)=\frac{\text{Var}(U)-\delta_{\text{min}}}{\delta_{\text{max}}-\delta_{\text{min}}},$$

with *δ*_*max*_ and *δ*_*min*_ being the maximum and the minimum values of *Var*(*U*) over all tests.

We point out that the heuristics in NHMC combines information on both the network structure, which affects *A*_*Y*_(·), and the hierarchical structure of the class, which is embedded in the computation of the distance, *d*(·,·) used in formula (5) and (2). We also note that the tree structure of the NHMC model makes it possible to consider different effects of the autocorrelation phenomenon at different levels of the tree model, as well as at different levels of the hierarchy (non-stationary autocorrelation). In fact, the effect of the class weights *ω*(*c*_*j*_) in Equation (3) is that higher levels of the tree will likely capture the regularities at higher levels of the hierarchy.

However, the efficient computation of distances according to Equation 3 is not straightforward. The difficulty comes from the need of computing *A*(*U*_1_) and *A*(*U*_2_)*incrementally*, i.e., from the statistics already computed for other partitions. Indeed, the computation of *A*(*U*_1_) and *A*(*U*_2_) from scratch for each partition would increase the time complexity of the algorithm by an order of magnitude and would make the learning process too inefficient for large datasets.

##### 

**Efficient computation of the heuristics** In our implementation, in order reduce the computational complexity, Equation (6) is not computed from scratch for each test to be evaluated. Instead, the first test to be evaluated is that which splits *U* in *U*_2_ ≠ *∅* and *U*_1_ ≠ *∅* such that |*U*_2_| is minimum (1 in most of cases, depending on the first available test) and *U*_1_ = *U* - *U*_2_. Only on this partition, Equation (6) is computed from scratch. The subsequent tests to be evaluated progressively move examples from *U*_1_ to *U*_2_. Consequently, *A*_*Y*_(*U*_1_),*A*_*Y*_(*U*_2_),*Var*(*U*_1_) and *Var*(*U*_2_) are computed incrementally by removing/adding quantities to the same values computed in the evaluation of the previous test.

*Var*(·) can be computed according to classical methods for incremental computation of variance. As regards *A*_*Y*_(·), its numerator (see Equation (5)) only requires distances that can be computed in advance. Therefore, the problem remains only for the denominator of Equation (5). To compute it incrementally, we consider the following algebraic transformations:

$$\begin{array}{ll} \;\sum_{u_{i}\in U}d{\left(L_{i},\bar{L_{U}}\right)}^{2} & =\underset{u_{i}\in U}{\sum }\overset{K}{\underset{k=1}{\sum }}\omega (c_{k}){\left(L_{i,k}-{\bar{L_{U}}}_{k}\right)}^{2}\\ & =\overset{K}{\underset{k=1}{\sum }}\omega (c_{k})\underset{u_{i}\in U}{\sum }{\left(L_{i,k}-{\bar{L_{U}}}_{k}\right)}^{2}\\ & =\overset{K}{\underset{k=1}{\sum }}\omega (c_{k})\underset{u_{i}\in U^{\prime }}{\sum }{\left(L_{i,k}-{\bar{L_{U^{\prime }}}}_{k}\right)}^{2}\\ & \;+(L_{t,k}-{\bar{L_{U^{\prime }}}}_{k})(L_{t,k}-{\bar{L_{U}}}_{k})\\ & =\underset{u_{i}\in U^{\prime }}{\sum }d{\left(L_{i},\bar{L_{U^{\prime }}}\right)}^{2}+(L_{t,k}-{\bar{L_{U^{\prime }}}}_{k})\\ & \;\times (L_{t,k}-{\bar{L_{U}}}_{k}) \end{array}$$

where *U* = *U*^′^ ∪ {*u*_*t*_} and $\bar{L_{U}}$ ( $\bar{L_{U^{\prime }}}$) is the average class vector computed on *U* (*U*^′^).

This allows us to significantly optimize the algorithm, as described in the following section.

##### 

**Time complexity** In NHMC, the time complexity of selecting a split test represents the main cost of the algorithm. In the case of a continuous split, a threshold *β* has to be selected for the continuous variable. If *N* is the number of examples in the training set, the number of distinct thresholds can be *N* - 1 at worst. Since the determination of candidate thresholds requires an ordering of the examples, its time complexity is *O*(*m* · *N* · *logN*), where *m* is the number of descriptive variables.

For each variable, the system has to compute the heuristic *h* for all possible thresholds. In general, this computation has time-complexity *O*((*N* - 1) · (*N* + *N* · *s*) · *K*), where *N* - 1 is the number of thresholds, *s* is the average number of edges for each node in the network, *K* is the number of classes, *O*(*N*) is the complexity of the computation of the variance reduction and *O*(*N* · *s*) is the complexity of the computation of autocorrelation.

However, according to the analysis reported before, it is not necessary to recompute autocorrelation values from scratch for each threshold. This optimization makes the complexity of the evaluation of the splits for each variable *O*(*N* · *s* · *K*). This means that the worst case complexity of creating a split on a continuous attribute is *O*(*m* · (*N* · *logN* + *N* · *s*) · *K*).

In the case of a discrete split, the worst case complexity (for each variable and in the case of optimization) is *O*((*d* - 1) · (*N* + *N* · *s*) · *K*), where *d* is the maximum number of distinct values of a discrete variable (*d* ≤ *N*). Overall, the identification of the best split node (either continuous or discrete) has a complexity of *O*(*m* · (*N* · *logN* + *N* · *s*) · *K*) + *O*(*m* · *d* · (*N* + *N* · *s*) · *K*), that is *O*(*m* · *N* · (*logN* + *d* · *s*) · *K*). This complexity is similar to that of CLUS-HMC, except for the *s* factor which equals *N* in the worst case, although such worst-case behavior is unlikely.

##### 

**Additional remarks** The relative influence of the two parts of the linear combination in Formula (6) is determined by a user-defined coefficient *α* that falls in the interval [0,1]. When *α* = 0, NHMC uses only autocorrelation, when *α* = 0.5, it weights equally variance reduction and autocorrelation, while when *α* = 1 it works as the original CLUS-HMC algorithm. If autocorrelation is present, examples with high autocorrelation will fall in the same cluster and will have similar values of the response variable (gene function annotation). In this way, we are able to keep together connected examples without forcing splits on the network structure (which can result in losing generality of the induced models).

Finally, note that the linear combination that we use in this article (Formula (6)) was selected as a result of our previous work on network autocorrelation for regression [12]. The variance and autocorrelation can also be combined in some other way (e.g., by multiplying them). Investigating different ways of combining them is one of the directions for our future work.

## Results and discussion

In this section, we present the evaluation of the system NHMC on several datasets related to predicting gene function in yeast. Before we proceed to presenting the empirical results, we provide a description of the datasets used and the experimental settings.

### Data sources

We use 12 datasets for gene function prediction in yeast (*Saccharomyces cerevisiae*) as considered by Clare and King [1], but with the class labels used by Vens et al. [4]^b^.

The **seq** dataset records sequence statistics that depend on the amino acid sequence of the protein for which the gene codes. These include amino acid frequency ratios, sequence length, molecular weight and hydrophobicity.

The **pheno** dataset contains phenotype data, which represent the growth or lack of growth of knock-out mutants that are missing the gene in question. The gene is removed or disabled and the resulting organism is grown with a variety of media to determine what the modified organism might be sensitive or resistant to.

The **struc** dataset stores features computed from the secondary structure of the yeast proteins. The secondary structure is not known for all yeast genes; however, it can be predicted from the protein sequence with reasonable accuracy, using Prof [24]. Due to the relational nature of secondary structure data, Clare and King [1] performed a preprocessing step of relational frequent pattern mining; the **struc** dataset includes the constructed patterns as binary attributes.

The **hom** dataset includes, for each yeast gene, information from other, homologous genes. Homology is usually determined by sequence similarity; here, PSI-BLAST [25] was used to compare yeast genes both with other yeast genes and with all genes indexed in SwissProt v39. This provided for each yeast gene a list of homologous genes. For each of these, various properties were extracted (keywords, sequence length, names of databases they are listed in, …). Clare and King [1] preprocessed these data in a similar way as the secondary structure data to produce binary attributes.

The **cellcycle, church, derisi, eisen, gasch1, gasch2, spo, exp** datasets include microarray yeast data [1]. Attributes for these datasets are real valued. They represent fold changes in gene expression levels.

We construct two versions of each dataset. The values of the descriptive attributes are identical in both versions, but the classes are taken from two different classification schemes. In the first version, they are from FUN ( http://mips.helmholtz-muenchen.de/proj/funcatDB/), a scheme for classifying the functions of gene products, developed by MIPS [26]. FUN is a tree-structured class hierarchy; a small part is shown in Figure 1(a). In the second version of the data sets, the genes are annotated with terms from the Gene Ontology (GO) [6] ( http://www.geneontology.org), which forms a directed acyclic graph instead of a tree: each term can have multiple parents (we use GO’s "is-a" relationship between terms). Only annotations from the first six levels were taken.

In addition, we use two protein-protein interaction networks (PPIs) for yeast genes. In particular, the networks BioGRID [27] and DIP [28] are used, which contain 323578 and 51233 interactions among 6284 and 7716 proteins, respectively. BioGRID stores physical and genetic interactions, DIP (Database of Interacting Proteins) stores and organizes information on binary protein-protein interactions that are retrieved from individual research articles.

The basic properties of the datasets in terms of the number of examples, number of attributes (features) and number of (hierarchically organized) classes are given in Table 1. For both networks, binary (0/1) weights are considered in NHMC. Exceptions are explicitly mentioned.

**Table 1:** Basic properties of the datasets

**Dataset**	**FUN**			**GO**		
	**#Instances**	**#Attributes**	**#Classes**	**#Instances**	**#Attributes**	**#Classes**
seq	3932	476	499	3900	476	4133
pheno	1592	67	455	1587	67	3127
struc	3838	19629	499	3822	19629	4132
hom	3848	47035	499	3567	47035	4126
cellcycle	3757	77	499	3751	77	4125
church	3779	550	499	3774	550	4131
derisi	2424	63	499	2418	63	3573
eisen	3725	79	461	3719	79	4119
gasch1	3764	172	499	3758	172	4125
gasch2	3779	51	499	3758	51	4131
spo	3703	79	499	3698	79	4119
exp	3782	550	499	3773	550	4131

We use 12 yeast (*Saccharomyces cerevisiae*) datasets (as considered by [1]) and two functional annotation (FUN and GO) schemes.

### Experimental setup

In the experiments, we deal with several dimensions: different descriptions of the genes, different descriptions of gene functions, and different gene interaction networks. We have 12 different descriptions of the genes from the Clare and King’ datasets [1] and 2 class hierarchies (FUN and GO), resulting in 24 datasets with several hundreds of classes each. Furthermore, we use BioGRID and DIP PPI networks for each of those. Moreover, for each dataset, we extracted the subset containing only the genes that are most connected, i.e., have at least 15 interactions in the PPI network (*highly connected genes*). We will focus on presenting the results for the datasets with GO annotations, while the results for the FUN versions of the datasets are given in the Additional files 3 and 4.

As suggested by Vens et al. [4], we build models trained on 2/3 of each data set and test on the remaining 1/3. The results reported in this paper are obtained using exactly the same splits as [4]. The subset containing genes with more than 15 connections uses the same 2/3-1/3 training-testing split. This is necessary in order to guarantee a direct comparison of our results with results obtained in previous work. However, in order to avoid problems due to randomization, we also performed experiments according to a 3-fold cross validation schema.

To prevent over-fitting, we used two pre-pruning methods: the minimal number of examples in a leaf (set to 5) and F-test pruning. The latter uses the F-test to check whether the variance reduction achieved after adding a test is statistically significant at a given level (0.001, 0.005, 0.01, 0.05, 0.1, 0.125). The algorithm takes as input a vector of significance levels/ *p*-values and by internal 3-fold cross-validation selects the one which leads to the smallest error.

Following Vens et al. [4], we evaluate the proposed algorithm by using as a performance metric the Average Area Under the Precision-Recall Curve ( $\bar{\text{AUPRC}}$), i.e., the (weighted) average of the areas under the individual (per class) Precision-Recall (PR) curves, where all weights are set to 1/|*C*|, with *C* the set of classes. The closer the $\bar{\text{AUPRC}}$ is to 1.0, the better the model is. A PR curve plots the precision of a classifier as a function of its recall. The points in the *PR* space are obtained by varying the value for the threshold *τ* from 0 to 1 with a step of 0.02. In the considered datasets, the positive examples for a given class are rare as compared to the negative ones. The evaluation by using *PR* curves (and the area under them), is the most suitable in this context, because we are more interested in correctly predicting the positive instances (i.e., that a gene has a given function), rather than correctly predicting the negative ones.

In order to evaluate the performance of the proposed NHMC algorithm, we compare it to CLUS-HMC (NHMC works just as CLUS-HMC when *α* = 1) which takes into account the attributes, as well as the hierarchical organization of classes, but does not consider network information. We also compare NHMC with the FunctionalFlow (FF) [29] and Hopfield (H) [30] approaches, which exploit the network information, but consider neither the attributes nor the hierarchical organization of classes. We report the results of NHMC with *α* = 0, when it uses only autocorrelation as a heuristic, and with *α* = 0.5, when it equally weights variance reduction and autocorrelation within the heuristic.

### Results for GO hierarchical multi-label classification

For each of the datasets, we report in Table 2 the $\bar{\text{AUPRC}}$ results obtained with NHMC (using *α* = 0.5 and *α* = 0.0), CLUS-HMC (i.e., NHMC with *α* = 1 which does not consider network information), FF and H (which do not consider the attributes and the hierarchical organization of classes). Two variants of each dataset are considered, one with all genes and the other with the subset of highly connected genes (with at least 15 connections). Furthermore, results for DIP and BioGRID are presented. For all genes, we also report 3-fold cross-validation $\bar{\text{AUPRC}}$ results in Table 3.

**Table 2:** The performance of NHMC and competitive methods in predicting gene function for different datasets and PPI networks

	**All genes**								
**Network**		**DIP**				**BioGRID**			
**Method**	**CLUS-HMC**	**NHMC**		**FF**	**H**	**NHMC**		**FF**	**H**
**Dataset**		***α* = 0**	***α* = 0.5**			***α* = 0**	***α* = 0.5**		
seq	0.023	0.032	0.030	0.004	0.003	0.011	0.011	0.006	0.006
pheno	0.019	0.016	0.016	0.001	0.001	0.016	0.016	0.003	0.002
struc	0.018	0.012	0.012	0.001	0.001	0.012	0.012	0.003	0.002
homo	0.040	0.013	0.013	0.000	0.000	0.012	0.012	0.001	0.002
cellcycle	0.019	0.287	0.288	0.004	0.003	0.012	0.012	0.006	0.006
church	0.014	0.015	0.012	0.003	0.002	0.012	0.012	0.006	0.006
derisi	0.017	0.015	0.017	0.004	0.003	0.044	0.317	0.006	0.006
eisen	0.030	0.024	0.024	0.005	0.003	0.015	0.334	0.006	0.008
gasch1	0.024	0.018	0.019	0.003	0.002	0.050	0.354	0.006	0.006
gasch2	0.020	0.021	0.021	0.004	0.003	0.012	0.012	0.006	0.006
spo	0.019	0.018	0.015	0.004	0.003	0.012	0.012	0.006	0.006
exp	0.023	0.017	0.016	0.003	0.002	0.012	0.012	0.006	0.006
Average:	0.022	0.041	0.040	0.003	0.002	0.018	0.093	0.005	0.005
	**Highly connected genes**								
**Network**		**DIP**				**BioGRID**			
**Method**	**CLUS-HMC**	**NHMC**		**FF**	**H**	**NHMC**		**FF**	**H**
**Dataset**		***α* = 0**	***α* = 0.5**			***α* = 0**	***α* = 0.5**		
seq	0.037	0.072	0.1	0.003	0.001	0.025	0.035	0.007	0.007
pheno	0.051	0.016	0.051	0.002	0.002	0.051	0.051	0.006	0.005
struc	0.078	0.078	0.078	0.001	0.002	0.078	0.078	0.003	0.003
homo	0.047	0.068	0.068	0.001	0.001	0.023	0.023	0.002	0.003
cellcycle	0.027	0.036	0.018	0.004	0.005	0.026	0.041	0.007	0.007
church	0.017	0.025	0.025	0.004	0.004	0.025	0.025	0.007	0.007
derisi	0.078	0.078	0.106	0.004	0.004	0.044	0.042	0.007	0.007
eisen	0.043	0.061	0.146	0.005	0.005	0.030	0.045	0.007	0.007
gasch1	0.051	0.094	0.095	0.004	0.005	0.050	0.046	0.007	0.007
gasch2	0.04	0.088	0.107	0.004	0.005	0.025	0.043	0.007	0.007
spo	0.04	0.078	0.09	0.004	0.005	0.026	0.035	0.007	0.007
exp	0.045	0.036	0.092	0.004	0.004	0.025	0.025	0.007	0.007
Average:	0.046	0.061	0.081	0.003	0.003	0.036	0.041	0.006	0.006

We use the 2/3-1/3 training-testing evaluation schema. We report the $\bar{\text{AUPRC}}$ of the CLUS-HMC (*α* = 1), NHMC (*α* = 0.5 and *α* = 0), FunctionalFlow (FF), and Hopfield (H) methods, when predicting gene function in yeast using GO annotations. We use 12 yeast (*Saccharomyces cerevisiae*) datasets (as considered by [1]). We consider all genes. Results for two PPI networks (DIP and BioGRID) are presented.

**Table 3:** The performance of NHMC and competitive methods in predicting gene function for different datasets and PPI networks

	**All genes**								
**Network**		**DIP**				**BioGRID**			
**Method**	**CLUS-HMC**	**NHMC**		**FF**	**H**	**NHMC**		**FF**	**H**
**Dataset**		***α* = 0**	***α* = 0.5**			***α* = 0**	***α* = 0.5**		
seq	0.030	0.025	0.025	0.003	0.002	0.022	0.022	0.004	0.006
pheno	0.021	0.018	0.019	0.002	0.001	0.018	0.018	0.004	0.002
struc	0.018	0.012	0.016	0.002	0.000	0.012	0.012	0.004	0.002
homo	0.040	0.013	0.031	0.001	0.001	0.013	0.013	0.002	0.002
cellcycle	0.017	0.297	0.273	0.004	0.002	0.013	0.013	0.006	0.006
church	0.017	0.013	0.012	0.003	0.002	0.012	0.012	0.006	0.006
derisi	0.018	0.022	0.021	0.004	0.002	0.039	0.315	0.006	0.006
eisen	0.025	0.020	0.020	0.004	0.002	0.021	0.335	0.006	0.008
gasch1	0.020	0.017	0.017	0.003	0.002	0.029	0.339	0.006	0.006
gasch2	0.019	0.020	0.018	0.004	0.002	0.015	0.016	0.006	0.006
spo	0.018	0.019	0.018	0.004	0.002	0.017	0.017	0.006	0.006
exp	0.020	0.017	0.017	0.002	0.002	0.018	0.018	0.006	0.006
Average:	0.022	0.041	0.041	0.003	0.002	0.019	0.094	0.005	0.005

We use the 3-fold cross-validation evaluation schema. The *average*$\bar{\text{AUPRC}}$ (estimated by 3-fold CV) of the CLUS-HMC (*α* = 1), NHMC (*α* = 0.5 and *α* = 0), FunctionalFlow (FF), and Hopfield (H) methods, when predicting gene function in yeast using GO annotations. We use 12 yeast (*Saccharomyces cerevisiae*) datasets. Results for two PPI networks (DIP and BioGRID) are presented.

On the datasets with all genes, the best results are overall obtained by NHMC with *α* = 0. For the DIP network, there is no clear difference between NHMC with *α* = 0 and NHMC with *α* = 0.5. Note that in the DIP network only a half of the genes have at least one connection to other genes.

On average, NHMC outperforms CLUS-HMC. The difference in performance is especially notable for some datasets, i.e., cellcycle when using the DIP network and derisi, eisen and gasch1 when using the BioGRID network. This indicates that while some form of autocorrelation on the GO labels is present in both networks (DIP and BioGRID), they provide different information. Exceptions are the *struc* and *hom* datasets. A possible explanation can be the high number of attributes, which may provide information redundant with respect to the information provided by the PPI networks. In this case, NHMC encounters the curse of dimensionality phenomenon [31].

The advantage of NHMC over CLUS-HMC comes from the simultaneous use of the hierarchy of classes and the PPI information in protein function prediction. It confirms the benefits coming from the consideration of autocorrelation during the learning phase. The tree structure of the learned models allows NHMC to consider different effects of autocorrelation at different levels of granularity. All these considerations are valid for both evaluation schemata we use, that is, the 2/3-1/3 training-testing split and the 3-fold cross-validation; schema.

The results obtained by using two functional linkage network (FLN) based algorithms, i.e., FunctionalFlow (FF) [29] and Hopfield (H) [30], are comparable between them, but are not comparable with those obtained by NHMC and CLUS-HMC. This is due to the different classification problem considered by FF and H, which does not take into account the attributes or the hierarchy of classes. Thus, NHMC and CLUS-HMC obtain far better accuracies than FF and H.

**Figure 2:**
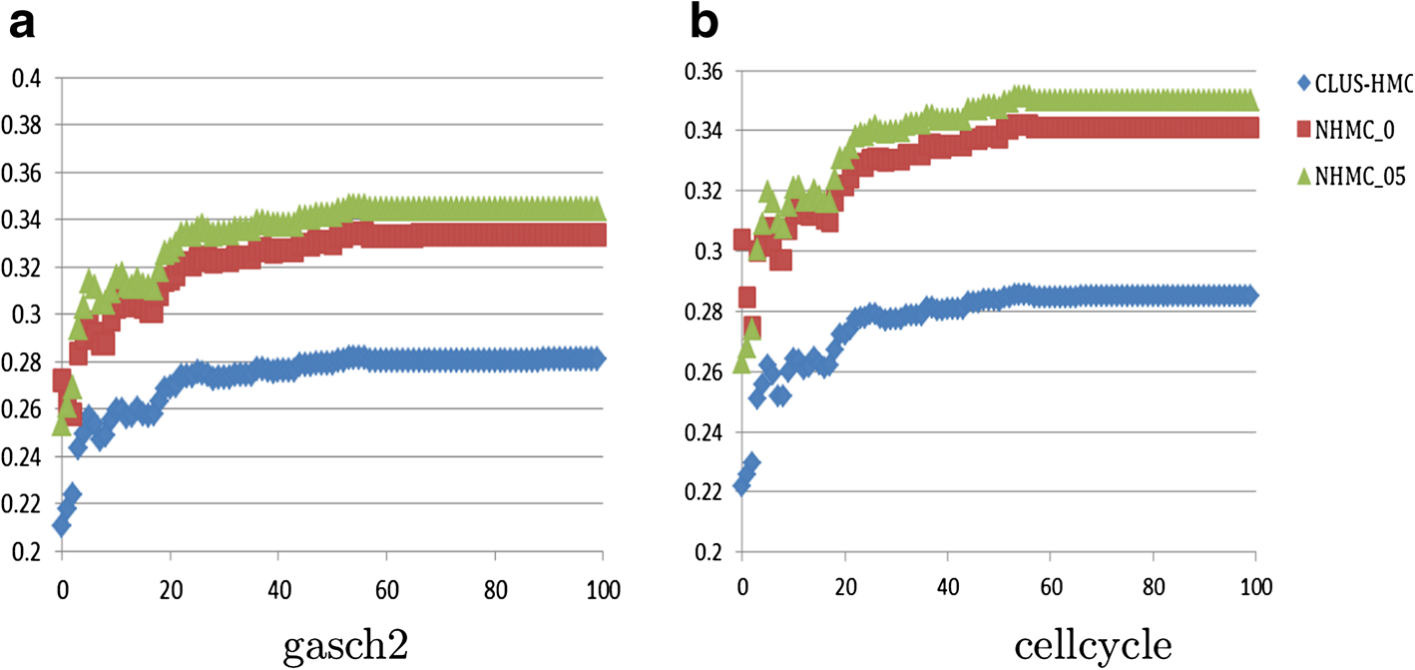
$\bar{\text{AUPRC}}$**distribution.** Comparison of the predictive models in terms of $\bar{\text{AUPRC}}$ learned by CLUS-HMC and NHMC (*α* = 0.5 and *α* = 0) from the most connected subsets of genes from the **(a)** gasch2 and **(b)** cellcycle datasets annotated with labels from the GO hierarchy. The horizontal axis gives the minimum relative number (in %) of interactions a gene must have in the DIP PPI network to be included in the testing data, whereas the vertical axis gives the model performance on the testing data in terms of the $\bar{\text{AUPRC}}$ values. At the far right (100 on the horizontal axis), we have the performance on the most-highly connected genes from the test set. At the far left (0 on the horizontal axis), we have the performance on all genes for the testing set.

As expected, when we use only highly connected genes for both training and testing, we obtain better performance. To investigate this effect in more detail, in Figure 2 we present the $\bar{\text{AUPRC}}$ results obtained by using CLUS-HMC and NHMC (with *α* = 0.5 and *α* = 0) for predicting GO annotations in the gasch2 (Figure 2(a)) and cellcycle (Figure 2(b)) datasets. The graphs depict the performance of the two models learned from highly connected genes from each dataset, for different portions of the genes from the testing data, ordered by the minimum number of connections of the gene in the DIP PPI network. Both CLUS-HMC and NHMC perform better when tested on highly connected genes only (far right in Figures 2(a) and 2(b)) as compared to being tested on all genes (far left). For both datasets, NHMC clearly performs better than CLUS-HMC for all subsets of the testing set. The difference in performance is less pronounced when all genes are considered (far left), but becomes clearly visible as soon as the genes with no or few connections are excluded, and are most pronounced for the most connected genes (far right). Note that no network information is used by NHMC about the testing set. This means that network information from the training set is sufficient to obtain good predictive models.

After considering the accuracy on the subset of highly connected genes (see Table 2), we turn our attention to the accuracy on the subset of weakly connected genes. This subset contains all genes that are not highly connected, i.e., have less than 15 interactions in the PPI network. In Table 4, we present the predictive performance of the models trained on the subset of highly connected genes and tested on the subset of remaining genes.

**Table 4:** The performance of NHMC and competitive methods in predicting gene function on weakly connected genes

	**15 connections**				**5 connections**			
**Dataset**	**CLUS-HMC**	**NHMC**	**FF**	**H**	**CLUS-HMC**	**NHMC**	**FF**	**H**
seq	0.014	0.014	0.001	0.001	0.033	0.042	0.007	0.007
pheno	0.018	0.051	0.001	0.001	0.033	0.027	0.005	0.007
struc	0.012	0.078	0.001	0.001	0.093	0.093	0.000	0.007
homo	0.012	0.023	0.001	0.001	0.149	0.149	0.003	0.007
cellcycle	0.015	0.015	0.001	0.001	0.041	0.023	0.007	0.007
church	0.013	0.025	0.001	0.001	0.031	0.022	0.007	0.007
derisi	0.015	0.015	0.000	0.001	0.024	0.026	0.007	0.007
eisen	0.020	0.020	0.000	0.001	0.039	0.040	0.007	0.002
gasch1	0.015	0.015	0.001	0.001	0.023	0.025	0.007	0.006
gasch2	0.018	0.023	0.001	0.001	0.028	0.028	0.007	0.007
spo	0.015	0.015	0.000	0.001	0.022	0.022	0.007	0.007
exp	0.015	0.015	0.001	0.001	0.026	0.044	0.007	0.003
Average:	0.015	0.026	0.001	0.001	0.045	0.045	0.006	0.006

We report the $\bar{\text{AUPRC}}$ of the CLUS-HMC (*α* = 1), NHMC (*α* = 0.5), FunctionalFlow (FF), and Hopfield (H) methods, when predicting gene function in yeast, using GO annotations and the BioGRID PPI network. The models are trained on the subset of highly connected genes and tested on the subset of weakly connected genes.

As expected, with a threshold of 15 connections (training on genes with more than 15 connections and testing on the remaining genes) the predictive accuracy decreases for all methods/models, but the reduction is the smallest for NHMC (28% on average) and the largest for the FLN-based algorithms (91% for FF and 84% for H on average). NHMC does not use the network information in the testing phase and its predictions are not affected much by this scenario, whereas the FLN-based algorithms are highly dependent on the network information and their accuracy decreases drastically. Moreover, the results obtained by using NHMC are better than those of CLUS-HMC on the subset of weakly connected genes. This means that NHMC can build better models because it uses both the hierarchy of classes and the network information, as compared to the models built by only using the hierarchy of classes (by CLUS-HMC) or by only using the network information (FF, H). In the case of a threshold of 5 connections, we have better results both for CLUS-HMC and NHMC. However, in this case, the advantage provided by the network information does not allow NHMC to outperform CLUS-HMC in all the datasets and the accuracies obtained by the two systems are very similar. This is mainly due to the noise present in the PPI networks (especially present in weakly connected genes) which works against the beneficial effect of the network^**c**^.

A different perspective of the results is presented in Table 5, where we compare the use of binary weights (used until now) with the use of a simple weighting that considers the number of times that an interaction is identified in the experiments. Results show that non-binary weighting is, in general, not beneficial. This behavior is observed not only for NHMC but also for FF and H. A possible explanation can be found in the quality of the weights which, according to the way they are generated, may introduce noise.

**Table 5:** Weighted features

	**Binary connections**			**Weighted connections**		
**Dataset**	**NHMC**	**FF**	**H**	**NHMC**	**FF**	**H**
seq	0.011	0.006	0.006	0.021	0.006	0.006
pheno	0.016	0.003	0.002	0.016	0.004	0.004
struc	0.012	0.003	0.002	0.093	0.002	0.003
homo	0.012	0.001	0.002	0.149	0.006	0.006
cellcycle	0.012	0.006	0.006	0.013	0.007	0.006
church	0.012	0.006	0.006	0.012	0.007	0.006
derisi	0.317	0.006	0.006	0.013	0.007	0.006
eisen	0.334	0.006	0.006	0.041	0.006	0.006
gasch1	0.354	0.006	0.006	0.016	0.007	0.006
gasch2	0.012	0.006	0.006	0.016	0.007	0.006
spo	0.012	0.006	0.006	0.016	0.007	0.006
exp	0.012	0.006	0.006	0.015	0.007	0.006
Average:	0.093	0.005	0.005	0.035	0.006	0.006

Binary vs. weighted connections: $\bar{\text{AUPRC}}$ of NHMC (*α* = 0.5) with weighted distances in the BioGRID PPI network.

A final remark concerns the sensitivity of NHMC to the presence of highly redundant features and how NHMC works in combination with a feature selection algorithm. With this goal in mind, we have used a spectral feature selection algorithm [32] with a normalized cut to select the 15%, 10%, 5% and 1% top ranked features for the two datasets characterized by a very large number of attributes, that is, struc and homo (19,629 and 47,035 attributes, respectively). The results obtained after learning models with reduced sets of features are reported in Table 6. They essentially show that NHMC is not affected by the high number of features. This can be explained by the top-down tree induction approach, which, at each internal node of the tree, selects the best attribute to be considered in the test and ignores the others, thus implicitly implementing an embedded feature selection [33] algorithm.

**Table 6:** The performance of NHMC in predicting gene function in combination with feature selection

	**Dataset**			
	**Struc**		**Homo**	
**Feature set**	**No. of features**	$\bar{\text{AUPRC}}$	**No. of features**	$\bar{\text{AUPRC}}$
All the features	19624	0.012	47034	0.012
Top 15%	2944	0.0115	7055	0.012
Top 10%	1962	0.0115	4703	0.012
Top 5%	981	0.0115	2351	0.012
Top 1%	196	0.0115	470	0.0115

$\bar{\text{AUPRC}}$ of NHMC (*α* = 0.5) with BioGRID PPI network for struc and homo datasets, when working on all the features, top 15% of the features, top 10% of the features, top 5% of the features and top 1% of the features. The datasets struc and homo are chosen because of their very high number of features as compared to the other datasets.

### Results for FUN Hierarchical Multi-label Classification

In addition to the experiments with GO annotations, we also perform experiments with FUN annotations. The results are reported in Additional file 3 and summarized here. They agree with results obtained with GO annotations, apart for the fact that when using all genes, NHMC performs comparably to CLUS-HMC. When working with highly connected genes, NHMC yields very good results, mainly better than CLUS-HMC, independently of the considered network.

We also compare the results of NHMC using FUN annotations to the results of additional methods from other studies. In particular, we compare our results to the results of three recent bio-inspired strategies which work in the HMC setting, but do not consider network information. The three methods are Artificial Neural Networks (HMC-LMLP), Ant Colony Optimization (hmAnt-Miner), and a genetic algorithm for HMC (HMC-GA) [34]. While the first algorithm is a 1-vs-all (it solves several binary classification problems) method based on artificial neural networks trained with the back-propagation algorithm, the latter two are methods that discover HMC rules. The algorithms are evaluated on 7 yeast FUN annotated datasets [1], using the same experimental setup we use for CLUS-HMC and NHMC, i.e., the setup proposed by Vens et al. [4].

In Additional file 4, we present the performance ( $\text{AU}\bar{\text{PRC}}$ results) obtained by using HMC-GA, HMC-LMLP, hmAnt-Miner and NHMC (*α* = 0.5) on 7 (from the above 12) FUN annotated datasets. NHMC outperforms all other methods by a wide margin. An exception is only the church dataset, for which NHMC performs worse than hmAnt-Miner. Note that the $\text{AU}\bar{\text{PRC}}$[34] measure used in this comparison is similar to $\bar{\text{AUPRC}}$, but uses weights that consider the number of examples in each class. We use $\text{AU}\bar{\text{PRC}}$ here to make our results easily comparable to the results obtained with the other three methods: The study by Cerri et al. [34] only gives their results in terms of $\text{AU}\bar{\text{PRC}}$.

### Using different PPI networks within NHMC

Although all PPI networks are frequently updated and maintained, many works have pointed out that the PPI networks are also very noisy (e.g., [35]). In the following, we argue that NHMC can be a valid tool for assessing the quality of network data in the context of exploiting information coming from PPI networks for gene function prediction. Before we compare the results obtained by NHMC using different PPI networks, we discuss some functional and topological properties of the 2 considered yeast PPI networks: DIP and BioGRID.

Table 7 shows the percentage of proteins that are covered (i.e., connected to other proteins within) by each of the PPI networks. While BioGRID has almost complete coverage, within the other network (DIP) only half of the proteins interact with other proteins. Next, Table 7 shows the percentage of function-relevant interactions. An interaction is considered to be function-relevant (with respect to a given hierarchy) if the two proteins involved in the interaction have at least one function (from the hierarchy) in common. In the 2 networks and across the 12 datasets, the degree of function relevance varies widely, i.e, 2%–33% of the observed interactions are function relevant. However, a closer look at the statistics reveals that the connections are much more function-relevant with respect to GO annotations than with respect to FUN annotations. This is largely due to the fact that GO contains a much larger number of functions. In addition, Table 7 gives the average degree of a node, i.e., the average number of neighbors that a node has in the network.

**Table 7:** Basic properties of the yeast PPI networks

**Dataset**	**% of connected genes**				**% of function-relevant interactions**				**Avg. degree of node**			
	**FUN**		**GO**		**FUN**		**GO**		**FUN**		**GO**	
	**DIP**	**BioGRID**	**DIP**	**BioGRID**	**DIP**	**BioGRID**	**DIP**	**BioGRID**	**DIP**	**BioGRID**	**DIP**	**BioGRID**
seq	46	96	46	97	8	8	15	8	7.09	7.09	7.15	54.97
pheno	46	98	46	99	6	11	16	11	3.53	27.67	17.57	27.75
struc	13	98	59	98	7	14	14	14	7.27	54.74	7.07	54.97
hom	45	97	48	14	7	16	14	16	7.22	54.301	7.79	58.57
cellcycle	72	99	47	99	2	17	17	16	7.36	55.63	7.38	55.72
church	46	99	46	99	15	16	13	15	7.35	56.21	7.39	56.28
derisi	72	100	73	100	7	17	11	16	11.17	84.43	11.19	84.64
eisen	35	65	35	65	9	19	19	17	4.68	32.47	4.69	32.52
gasch1	47	99	47	99	9	17	19	16	7.41	55.83	7.42	55.92
gasch2	47	98	47	99	7	17	17	16	7.35	55.62	7.39	55.93
spo	48	99	48	99	3	13	17	16	7.31	55.27	7.32	55.35
exp	46	99	46	99	15	16	39	15	7.35	56.16	7.36	56.3

The percentage of connected genes, the percentage of function-relevant interactions and the average degree of nodes for 2 different PPI networks (DIP [28] and BioGRID [27].

Having described some of the characteristics of the different PPI networks used in this study, we can now proceed with the comparison of the results obtained by using these networks within NHMC. Comparing the NHMC results obtained with DIP and BioGRID for GO annotations (Table 2), we see that DIP leads to higher $\bar{\text{AUPRC}}$ results. The "% of function-relevant interactions" is on average higher for DIP, even though the number of connected genes in BioGRID is twice as high as the one in DIP (see Table 7). This indicates that BioGRID, although denser than DIP, does not provide the same quantity/type of information for the gene function prediction task as DIP. Similar conclusions hold for predicting gene functions within the FUN annotation scheme, as evident from Additional file 3.

Finally, comparing the results in Tables 2 and 3 and the table reported in Additional file 3, we see that, although for GO we have a higher "% of function-relevant interactions" in both the DIP and the BioGRID networks, the learning task for GO is more complex than that for FUN. This is primarily due to the significantly higher number of classes in GO. This explains the better $\bar{\text{AUPRC}}$ values for FUN in comparison to those for GO, when comparing the results of NHMC on the same dataset and using the same network.

### Related work

Many machine learning approaches that tackle the problem of protein function prediction have been proposed recently (starting from the seminal work by Pavlidis et al [36]). A review of the plethora of existing methods can be found in [3,37]. In this section, we will only discuss related works which are close to ours along two dimensions: using hierarchical annotations [8,38,39] and using network information [5,40,41].

#### HMC for predicting gene function

Our work builds on the foundations by Vens et al. [4], where the hierarchical constraint is enforced by the algorithm CLUS-HMC that learns predictive clustering trees (PCTs) for HMC. Recently, Cerri et al. [34] applied a genetic algorithm (HMC-GA) to solve the HMC problem. In their method, the antecedents of decision rules evolve with a biased fitness function towards rules with high example coverage. The method also removes from the training set examples already covered by the generated rules. Valentini [42] developed the *true-path rule* (*hierarchical constraint*) ensemble learner for genome-wide gene function prediction, where positive (negative) probabilistic predictions for a node transitively influence the ancestors (descendants) of the node. While [4] and [34] ignore information coming from relationships among examples, [42] exploits PPI networks. However, the considered information is limited to binary (input) attributes, which describe the interaction of a gene with specific other genes.

#### Using PPI networks in predicting gene function

Recent reviews of the latest techniques that use PPI data for protein function prediction [2,37,43,44] suggest that it is possible to distinguish two major approaches. The first one explores direct annotation schemes and infers the function of a protein based on its connections in the network. The second one, module-assisted, first identifies modules of related proteins and then annotates each module. The first approach is followed by Letovsky and Kasif [45], who apply a Markov random field model. There, a node’s label probability is entirely a function of its neighbors’ states. In addition, Karaoz et al. [30] (similarly to Vazquez et al. [46]) presented a functional linkage network (FLN) based algorithm (Hopfield), inspired by discrete-state Hopfield Networks as used in physics, for predicting the functions of genes. The method constructs a graph, whose nodes are genes and edges connect genes that may share the same function, by integrating gene expression data, protein-protein interactions and protein-DNA binding data. FunctionalFlow [29] generalizes the *guilt-by-association* principle to groups of proteins that may interact with each other physically. The algorithm annotates nodes as an infinite reservoir of functional flow. Initially, each node with known GO functional annotation is a "source" for that function. In each round, "function" flows along the weighted edges of the graph. In Nariai et al. [47], nodes in the graph are genes and edges represent evidence for functional similarity based on gene expression data, protein-protein interactions and protein-DNA binding data. Some form of autocorrelation, limited to directly connected nodes, is considered. However, the above approaches [29,30,45-47] do not consider the hierarchy of categories.

The second approach attempts to define the relationship between the PPI network topology and biological protein function. Milenkovic and Przulj [48] relate the PPI network structure to protein complexes. They group proteins by considering local topology of the PPI network and show that these protein groups belong to the same protein complexes, perform the same functions, are localized in the same compartments, and have the same tissue expressions. The work by Borgwardt et al. [49] uses graph kernels that measure the similarity between graphs and learns a support vector machine classifier for protein function prediction. The graph model combines sequential, structural and chemical information about proteins. Gillis and Pavlidis [20] recommend to test the effect of critical edges (based on node degree) when assessing network quality using GBA-like approaches. Tao et al. [50] modified the traditional k-NN classification algorithm to consider the semantic similarity between functional classes when predicting the functions of genes based on GO annotations. Pandey et al. [51] used the same algorithm and the same similarity measure, but using a different definition that also includes the similarity between the sets of functional labels of two proteins. The difference between these two approaches [50,51] and our approach is that they use GO to identify the distances, while we use PPI networks to identify relationships and classify genes according to GO.

##### 

**What distinguishes our work from related work** As discussed above, many approaches exist for gene function prediction, some take into account PPI networks and some take into account the hierarchical organization of annotation schemes. Most approaches in the first category do not consider the hierarchical constraint directly, but rather in a post-processing phase. Most approaches in the second category do not explicitly consider the effect of network autocorrelation of gene function. Moreover, none of them, takes into account simultaneously the hierarchical constraint and network autocorrelation in predicting gene function.

In contrast to related works, in general and the related work described above in particular, our approach considers both the network autocorrelation that arise in the PPI networks and the hierarchical organization of the annotation schemes. In general, our approach NHMC performs better than the approaches that consider only the network or only the hierarchy of classes. This was clearly demonstrated above through the empirical comparison of NHMC with FunctionalFlow and Hopfield algorithms, on one hand, and with CLUS-HMC, HMC-GA, HMC-LMLP and hmAnt-Miner on the other hand.

Moreover, the network setting that we use in this work is different from that used in other studies where the network is not considered at all (although PPI networks give valuable information) or is considered in tight connection to the data so that predictions can be made only for genes for which interactions with other genes are known. In our approach, the network structure is considered only during the training phase (model creation) and is disregarded during the testing phase. This key feature of the proposed approach is especially attractive from a practical perspective when the function we want to predict of new genes for which interactions with other genes may exist, but are not known or still need to be confirmed.

## Conclusion

In this work, we address the problem of learning to predict gene/protein function by exploiting their individual properties as well as their interactions (as captured in protein-protein interaction / PPI networks). In contrast to most existing approaches, which use only one of the two sources (properties or networks), we use both. Moreover, we only use the network information in the training phase and can thus make predictions for genes/proteins whose interactions are yet to be investigated.

We view the problem of gene/protein function predictions as a problem of hierarchical multi-label classification, where instances may belong to multiple classes and the relationships between the classes are hierarchical. We also consider relations among the instances, i.e., interactions of the proteins within a PPI network: These relations introduce autocorrelation and violate the assumption that instances are independently and identically distributed (i.i.d.), which underlines most machine learning algorithms. While the consideration of these relations introduces additional complexity into the learning process, it can also bring substantial benefits.

The major contributions of our paper are as follows. First, we formulate the problem of hierarchical multi-label classification in a network setting: To the best of our knowledge, the HMC task of structured-output prediction has not been considered in a network setting before. Second, we define the notion of autocorrelation for such a setting and introduce an appropriate measure of network autocorrelation for HMC. Third, we develop a machine learning method for solving the task of HMC in a network setting, which successfully exploits the network information (via autocorrelation) during the learning phase.

Finally, we perform an extensive empirical evaluation of the proposed machine learning method for HMC in a network setting on the task of predicting yeast gene/protein function in a PPI network context. We use a variety of datasets describing genes in different ways, two functional annotation schemes (GO and FUN) and two different PPI networks (DIP and BioGRID). In sum, the results of the evaluation show that our method, which uses both gene/protein properties and network information, yields better performance than the methods using each of these two sources separately: Overall, this holds across the different gene descriptions, annotation schemes, and different networks. The benefits of using the network information are diminished (more difficult to reap) when the genes/proteins are described with a very large number of features, i.e., under the curse of dimensionality.

More specifically, the properties of the PPI network used have a strong influence on the overall performance. Best results are achieved when the PPI network is reasonably dense and contains a large proportion of function-relevant interactions. The DIP and BioGRID networks rate best in this respect and lead to most notable improvements in performance when used within our method. It is worth noting here that networks (such as BioGRID) are nowadays often tuned for function-relevance with respect to the commonly used Gene Ontology (GO) annotation scheme.

Finally, note that the use of network information improves the accuracy of gene function prediction not only for highly connected genes, but also for genes with only a few connections (or none at all). Note also that we do not need information on the network around a gene when we want to predict the function for a new gene: This is important for genes that are not well known (especially in terms of interactions). In such a case, we can expect better predictions from the models learned by our approach, regardless of whether the gene is well connected or only weakly connected to other genes and regardless of the fact whether its connectivity is known or unknown.

We will explore several avenues for development and evaluation of our approach in further work. In terms of development, we plan to consider different ways for combining variance reduction and autocorrelation within the search heuristic used in our approach. In terms of evaluation, we plan to use additional datasets and networks. This will include new datasets for organisms other that yeast and networks based on sequence similarity (usually implying homology) among genes, as well as more recent function labels for the presently considered datasets. In the context of the latter, we will consider additional networks with non-binary weights that reflect the strength of the connections within the network.

## Availability of supporting data

**Project Name:** NHMC

**Project Home Page:**http://kt.ijs.si/daniela_stojanova/NHMC/

**Available resources:** NHMC software and user manual, and the datasets.

## Endnotes

^**a**^ Note that the tree structure induced by CLUS-HMC does not directly reflect the hierarchy of classes. For this reason, in this paper, we will distinguish between the terms *hierarchy* (of classes) and *tree* (model for HMC).

^**b**^ The function labels were downloaded in 2008. On one side, this facilitates comparison with previous work. On the other side, it is possible that results with new labels would be slightly different.

^**c**^ Obviously, the considered case is an extreme situation where the evaluation suffers from the non-random distribution of the examples.

## Competing interests

The authors declare that they have no competing interests.

## Authors’ contributions

SD conceived of the study, which was primarily carried out by DS and MC. DS designed and implemented NHMC, while DS and MC carried out the experimental evaluation of NHMC and other methods on the different tasks of gene function prediction. DS, MC, SD and DM contributed to the manuscript drafting and finalization. MC, SD and DM participated in the design of the study. SD and DM participated in the BioGRID: a general repository for interaction datasets 2006.coordination of the study. All authors read and approved the final manuscript.

## Supplementary Material

Additional file 1 Algorithm 2: Pseudo-code of the CLUS-HMC algorithm for top-down induction of HMC trees. Click here for file

Additional file 2 **Geary’s** ***C* for Spatial Regression.** Click here for file

Additional file 3 The performance of the models for predicting FUN annotations. Click here for file

Additional file 4 The performance of NHMC and other methods in predicting FUN annotations of yeast genes. Click here for file
