# Supplementary material for: Using PPI network autocorrelation in hierarchical multi-label classification trees for gene function prediction
Source: BMC Bioinformatics. 2013 Sep 26;14:285. doi: 10.1186/1471-2105-14-285 (PMC3850549; doi:10.1186/1471-2105-14-285)
Supplement: Additional file 1 — Algorithm 2: Pseudo-code of the CLUS-HMC algorithm for top-down induction of HMC trees. [file 1471-2105-14-285-S1.pdf]

Additional file 1 — Algorithm 2: Pseudo-code of the CLUS-HMC algorithm for top-down induction of HMC trees

Algorithm 2 Top-down induction of CLUS-HMC

procedure CLUSHMC(G, U) **returns** tree
if stop(U) **then**
  return leaf(Prototype(U))
else
  $(t^*, h^*, \mathcal{P}^*) = (null, 0, \emptyset)$
  for each possible Boolean test t according to the values of attribute X in dataset U **do**
    $\mathcal{P} = \{U_1, U_2\}$ partition induced by t on U
    $h = Var'(U) - \frac{|U_1| \cdot Var'(U_1) + |U_2| \cdot Var'(U_2)}{|U|})$
    if $(h > h^*)$ **then**
      $(t^*, h^*, \mathcal{P}^*) = (t, h, \mathcal{P})$
    end if
  end for
  $tree_1$ = CLUSHMC(U_1)
  $tree_2$ = CLUSHMC(U_2)
  return node($t^*, tree_1, tree_2$)
end if
