# Supplementary material for: Using PPI network autocorrelation in hierarchical multi-label classification trees for gene function prediction
Source: BMC Bioinformatics. 2013 Sep 26;14:285. doi: 10.1186/1471-2105-14-285 (PMC3850549; doi:10.1186/1471-2105-14-285)
Supplement: Additional file 2 — Geary’s C for Spatial Regression. [file 1471-2105-14-285-S2.pdf]

Additional file 2 — Geary's C for Spatial Regression

For the task of (single-target) spatial regression [12], where a real-valued response variable Y has to be predicted, many autocorrelation measures have been defined. Among these measures, the Global Geary's C [21] is one of the most well-known. It is defined as follows:

$$C_Y = \frac{(N-1)\sum_i \sum_j w_{ij}(y_i - y_j)^2}{2\sum_{i,j} w_{ij} \sum_i (y_i - \overline{y})^2} \tag{8}$$

where N is the number of spatial objects indexed by i and j; y_i and y_j are the values of the response variable Y observed for the spatial objects o_i and o_j, respectively; $\overline{y}$ is the overall mean of Y; and $w_{ij}, i, j = 1, \ldots, N$ are the values of a $N \times N$ matrix of spatial weights. The values of C_Y vary in the interval $[0, 2]$, where 0 indicates perfect positive autocorrelation (clustered pattern in the data distribution), 1 indicates no autocorrelation (random distribution of the data) and 2 indicates perfect negative autocorrelation (dispersed pattern in the data distribution).
