# Supplementary material for: Using PPI network autocorrelation in hierarchical multi-label classification trees for gene function prediction
Source: BMC Bioinformatics. 2013 Sep 26;14:285. doi: 10.1186/1471-2105-14-285 (PMC3850549; doi:10.1186/1471-2105-14-285)
Supplement: Additional file 3 — The performance of the models for predicting FUN annotations. [file 1471-2105-14-285-S3.pdf]

Additional file 3 — The performance of the models for predicting FUN annotations

The $\overline{AUPRC}$ of CLUS-HMC ($\alpha = 1$) and NHMC ($\alpha = 0.5$ and $\alpha = 0$) when predicting gene function in yeast in terms of FUN annotations. We use 12 yeast (*Saccharomyces cerevisiae*) datasets (as considered by [1]). We consider all genes as well as the subset of highly connected genes (with more than 15 connections). The BioGRID and DIP PPI networks for yeast genes are used.

	All genes					Highly connected genes				
Method	CLUS-HMC	DIP		BioGRID		CLUS-HMC	DIP		BioGRID	
Dataset		$\alpha = 0.5$	$\alpha = 0$	$\alpha = 0.5$	$\alpha = 0$		$\alpha = 0.5$	$\alpha = 0$	$\alpha = 0.5$	$\alpha = 0$
seq	0.059	0.054	0.053	0.056	0.062	0.051	0.094	0.130	0.051	0.058
pheno	0.036	0.035	0.028	0.028	0.028	0.068	0.333	0.333	0.064	0.064
struc	0.030	0.020	0.020	0.020	0.020	0.093	0.088	0.093	0.093	0.093
homo	0.073	0.020	0.023	0.020	0.020	0.149	0.088	0.088	0.088	0.088
cellcycle	0.032	0.030	0.037	0.020	0.029	0.047	0.098	0.125	0.034	0.050
church	0.029	0.020	0.020	0.020	0.020	0.041	0.091	0.091	0.033	0.033
derisi	0.027	0.028	0.025	0.020	0.026	0.048	0.098	0.119	0.048	0.093
eisen	0.047	0.042	0.025	0.024	0.037	0.067	0.147	0.183	0.039	0.069
gasch1	0.036	0.040	0.032	0.033	0.036	0.060	0.103	0.124	0.062	0.063
gasch2	0.034	0.034	0.027	0.025	0.028	0.037	0.108	0.112	0.051	0.052
spo	0.030	0.029	0.025	0.020	0.027	0.044	0.049	0.134	0.034	0.051
exp	0.040	0.030	0.025	0.020	0.028	0.067	0.091	0.132	0.029	0.027
Average:	0.039	0.032	0.028	0.026	0.030	0.064	0.116	0.139	0.052	0.062
