# Supplementary material for: Using PPI network autocorrelation in hierarchical multi-label classification trees for gene function prediction
Source: BMC Bioinformatics. 2013 Sep 26;14:285. doi: 10.1186/1471-2105-14-285 (PMC3850549; doi:10.1186/1471-2105-14-285)
Supplement: Additional file 4 — The performance of NHMC and other methods in predicting FUN annotations of yeast genes. [file 1471-2105-14-285-S4.pdf]

Additional file 4 — The performance of NHMC and other methods in predicting FUN annotations of yeast genes

The $AU\overline{PRC}$ of models learned by HMC-GA, HMC-LMLP, hmAnt-Miner and NHMC (using $\alpha = 0.5$ and the DIP PPI network), for 7 FUN annotated yeast datasets, as used in Cerri at al. [33].

Dataset/Method	HMC-GA	HMC-LMLP	hmAnt-Miner	NHMC ($\alpha = 0.5$)
pheno	0.148	0.085	0.162	0.241
cellcycle	0.150	0.144	0.154	0.173
church	0.149	0.140	0.168	0.152
derisi	0.152	0.138	0.161	0.172
eisen	0.165	0.173	0.180	0.196
gasch2	0.151	0.132	0.163	0.186
spo	0.151	0.139	0.174	0.181
